# Effectiveness of a Web-Based Self-Help Intervention for Symptoms of Depression, Anxiety, and Stress: Randomized Controlled Trial

**DOI:** 10.2196/jmir.954

**Published:** 2008-03-25

**Authors:** Annemieke van Straten, Pim Cuijpers, Niels Smits

**Affiliations:** ^1^Vrije Universiteit AmsterdamDepartment of Clinical PsychologyAmsterdamThe Netherlands

**Keywords:** Bibliotherapy, psychotherapy, problem-solving therapy, depression, anxiety, stress, randomized controlled trial

## Abstract

**Background:**

Self-help therapies are often effective in reducing mental health problems. We developed a new Web-based self-help intervention based on problem-solving therapy, which may be used for people with different types of comorbid problems: depression, anxiety, and work-related stress.

**Objective:**

The aim was to study whether a Web-based self-help intervention is effective in reducing depression, anxiety, and work-related stress (burnout).

**Methods:**

A total of 213 participants were recruited through mass media and randomized to the intervention (n = 107) or a waiting list control group (n = 106). The Web-based course took 4 weeks. Every week an automated email was sent to the participants to explain the contents and exercises for the coming week. In addition, participants were supported by trained psychology students who offered feedback by email on the completed exercises. The core element of the intervention is a procedure in which the participants learn to approach solvable problems in a structured way. At pre-test and post-test, we measured the following primary outcomes: depression (CES-D and MDI), anxiety (SCL-A and HADS), and work-related stress (MBI). Quality of life (EQ-5D) was measured as a secondary outcome. Intention-to-treat analyses were performed.

**Results:**

Of the 213 participants, 177 (83.1%) completed the baseline and follow-up questionnaires; missing data were statistically imputed. Of all 107 participants in the intervention group, 9% (n = 10) dropped out before the course started and 55% (n = 59) completed the whole course. Among all participants, the intervention was effective in reducing symptoms of depression (CES-D: Cohen’s *d* = 0.50, 95% confidence interval (CI) 0.22-0.79; MDI: *d* = 0.33, 95% CI 0.03-0.63) and anxiety (SCL-A: *d* = 0.42, 95% CI 0.14-0.70; HADS: *d* = 0.33, 95% CI 0.04-0.61) as well as in enhancing quality of life (*d* = 0.31, 95% CI 0.03-0.60). Moreover, a higher percentage of patients in the intervention group experienced a significant improvement in symptoms (CES-D: odds ratio [OR] = 3.5, 95% CI 1.9-6.7; MDI: OR = 3.7, 95% CI 1.4-10.0; SCL-A: OR = 2.1, 95% CI 1.0-4.6; HADS: OR = 3.1, 95% CI 1.6-6.0). Patients in the intervention group also recovered more often (MDI: OR = 2.2; SCL-A: OR = 2.0; HADS < 8), although these results were not statistically significant. The course was less effective for work-related stress, but participants in the intervention group recovered more often from burnout than those in the control group (OR = 4.0, 95% CI 1.2-13.5).

**Conclusions:**

We demonstrated statistically and clinically significant effects on symptoms of depression and anxiety. These effects were even more pronounced among participants with more severe baseline problems and for participants who fully completed the course. The effects on work-related stress and quality of life were less clear. To our knowledge, this is the first trial of a Web-based, problem-solving intervention for people with different types of (comorbid) emotional problems. The results are promising, especially for symptoms of depression and anxiety. Further research is needed to enhance the effectiveness for work-related stress.

**Trial Registration:**

International Standard Randomized Controlled Trial Number (ISRCTN) 14881571

## Introduction

It has been convincingly demonstrated that self-help therapies are effective in reducing mental health problems [1-5]. A self-help therapy can be defined as a standardized psychological treatment that the patient works through independently at home [6]. It is commonly delivered in book format, in which case it is called “bibliotherapy.” However, the therapy can also be delivered through other media, such as CD-ROMs, television programs, or videotapes. In recent years, self-help has been increasingly offered through the Internet [5,7,8]. Web-based self-help may be an effective and inexpensive alternative to more traditional therapies, especially since the majority of persons in the general population with a mental health disorder (an estimated 65%) do not receive help from any professional mental health services [9,10].

The self-help therapies that are currently available have all been developed for patients with a specific disorder, such as depression, panic disorder, social phobia, general anxiety disorder, or posttraumatic stress disorder, and most are based on cognitive behavioral therapy. Problem-solving therapy, a brief form of psychotherapy where patients identify their most immediate problems and ways of regaining control over them, are not limited to one specific disorder and may be effective in several problem areas. Face-to-face problem-solving therapies have been shown to be effective in depression [11,12] and several other mental health problems [13-15]. We know that at least one Web-based cognitive behavioral therapy includes a problem-solving module (MoodGYM) [16,17], but as far as we know, there is no Web-based therapy that uses problem solving as the core element. Therefore, we decided to develop a new, problem-focused, generic self-help method for multiple mental health problems that could be applied through the Internet.

As a general framework for the intervention, we used the model developed by Bowman and colleagues, which is based on problem-solving therapy [18,19]. The general idea of this intervention, which is called self-examination therapy, is that participants learn to regain control over their problems and lives by (1) determining what really matters to them, (2) investing energy only in those problems that are related to what matters, (3) thinking less negatively about the problems that are unrelated and, (4) accepting those situations that cannot be changed. This method has been found to be effective in several studies in the United States [14,19,20]. We used the self-examination therapy as a framework for our intervention but translated it into Dutch, elaborated on it, and added information and exercises. We built a website for this intervention and developed a system for email support.

The aim of this study was to determine the effectiveness of this Web-based generic treatment method for participants with depression, anxiety, and work-related stress.

## Methods

### Recruitment of Participants

**Figure 1:**
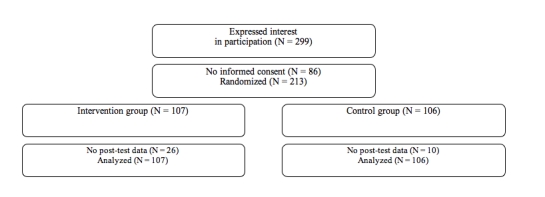
Flowchart of participants

We recruited participants through advertisements about Internet self-help treatment for symptoms of depression, anxiety, and work-related stress placed in local and national newspapers. We aimed at including 200 participants in order to be able to demonstrate moderate effects of *d* = 0.40 while using a power (1 − β) of 80% and an alpha of .05. We were contacted through email by 299 people (Figure 1). These 299 potential participants received an information booklet and an informed consent form by post as well as a baseline questionnaire through the Internet. All 213 individuals who returned the informed consent and the baseline questionnaire were included. No inclusion or exclusion criteria were used because the intervention was aimed at the general population. Enrollment took place between November 30 and December 20, 2005. The study was approved by the Medical Ethical Committee of the Vrije Universiteit Medical Center, Amsterdam, The Netherlands.

### Intervention

The intervention was Web-based (see Multimedia Appendix for screenshots). Participants were provided with a username and password to access the website. Every week an automated email was sent to the participants to explain the contents and exercises for the coming week. All the information as well as the exercise forms could also be downloaded from the website in case participants preferred to read the information on paper. Master’s level psychology students, trained and supervised by the authors (PC, AvS), offered feedback on the completed exercises. This feedback was not therapeutic but was directed at mastering the proposed problem-solving strategies. For a participant completing the course, the total time spent by the psychology students on feedback was approximately 45 minutes. The course takes 4 weeks.

The intervention consists of three steps:

1. Participants describe what really matters to them.
2. Participants write down their current worries and problems and categorize them into three types: (a) unimportant problems (problems unrelated to the things that matter to them), (b) problems that can be solved, and (c) problems that cannot be solved (eg, the loss of a loved one).
3. Participants make a plan for the future in which they describe how they will try to accomplish those things that matter most to them.

The second step is the most important of the intervention. For each of the three types of problem (ie, a, b, and c), a different strategy is proposed to cope with it. For the solvable problems (ie, b), we propose the following procedure: (1) write a clear definition of the problem, (2) generate multiple solutions to the problem, (3) select the best solution, (4) work out a systematic plan for this solution, (5) carry out the solution, and (6) evaluate as to whether the solution has resolved the problem.

### Design

All participants were randomly assigned to either the self-help course or a waiting list. Questionnaires were sent before the start of the course and 5 weeks later, after the intervention group had finished. Thereafter, the participants in the waiting list group could complete the course.

### Randomization

Randomization took place 1 week before the start of the intervention. We used block randomization with blocks of 10. The randomization scheme was derived by computer and carried out by an independent researcher. All participants were informed by email about the randomization outcome.

### Measures

Depressive symptoms were measured with the Center for Epidemiological Studies Depression Scale (CES-D) [21] and the Major Depression Inventory (MDI) [22]. The CES-D is a 20-item, self-report questionnaire on feelings of depression; its total score ranges from 0 (no depressive symptoms at all) to 60 (many depressive symptoms). The MDI contains 12 items that are used to calculate the scores on the 10 ICD-10 (International Statistical Classification of Diseases and Related Health Problems, 10th Revision) symptoms of depression. Each of the 10 symptoms is scored on a scale from 0 (at no time) to 5 (all of the time). The total score is calculated by adding all the items, and thus ranges from 0 to 50. Based on the symptom scores, it is also possible to determine the presence or absence of major depression according to the DSM-IV (Diagnostic and Statistical Manual of Mental Disorders, 4th Edition) criteria.

Symptoms of anxiety were measured with the seven anxiety questions of the Hospital Anxiety and Depression Scale (HADS) [23] and the anxiety section of the Symptom Checklist (SCL-A) [24]. The total score of the HADS varies from 0 (no complaints of anxiety) to 21 (many complaints of anxiety). The SCL-A consists of 10 questions, and the total score ranges from 10 (no complaints of anxiety) to 50 (many complaints of anxiety).

Work-related stress was measured with the Dutch version of the Maslach Burnout Inventory (MBI) [25], which contains three subscales: (1) emotional exhaustion (MBI-EE), 5 items; (2) depersonalization (MBI-DP), 4 items; and (3) personal accomplishment (MBI-PA), 6 items. Each item is scored on a scale from 0 to 6, and subscale scores are calculated by adding the item scores and dividing this subscale total score by the number of items. For MBI-EE and MBI-DP, a higher score indicates more work-related stress, while a high MBI-PA score indicates less work-related stress. Individuals can be considered burnt out when they report high MBI-EE (≥ 2.2) in combination with high MBI-DP (≥ 2.0) or low MBI-PA (≤ 3.66) [26].

Quality of life was assessed with the EuroQoL questionnaire (EQ-5D) [27]. The EQ-5D consists of 5 items (mobility, self-care, usual activities, pain/discomfort, and anxiety/depression), each of which is rated as causing “no problems,” “some problems,” or “extreme problems.” The EQ-5D can thus describe 486 unique health states. Each of these health states has been empirically valued between 0 (poor health) and 1 (perfect health). The scores of our respondents were weighted with these values to derive a single summary index score.

### Analyses

#### Missing Values

All analyses were performed on the intention-to-treat sample. Pre-test data were available for all participants. Missing values of post-test nonresponders (17%, 36/213) were handled by using multiple imputation procedure NORM [28] in statistical package R. In this procedure, missing data are imputed by regression analyses using available baseline data (demographics as well as data on baseline severity) from the responders as well as the nonresponders. This means that not every nonresponder received the same post-test score, but the post-test score was dependent on the particular characteristics as defined by baseline (eg, gender, age). This regression analyses was then repeated five times. The effectiveness analyses were then performed on each of the five resulting data files, and the five estimates were combined into a single overall estimate using the multiple imputation inference rules of Rubin [29]. This yielded proper *P* values and confidence intervals for the estimates. All reported *P* values are two-tailed.

#### Effectiveness

Effectiveness was calculated in three ways: (1) analyzing mean improvement scores, (2) calculating the proportion of participants who made significant improvements, and (3) calculating the proportion of participants who recovered. Each will be described in more detail below.

##### Mean Improvement Scores

The magnitude of the effect of the intervention (Cohen’s *d*) was calculated by subtracting the post-test mean score of the control group (M_c_) from the post-test mean score of the intervention group (M_i_) and dividing the result by the pooled standard deviation (SD_ic_). A Cohen’s *d* of 0.5 thus indicates that the mean of the intervention group is half a standard deviation larger than the mean of the control group. Values of *d* from 0.56 to 1.2 can be assumed to be large, 0.33 to 0.55 are moderate, and 0 to 0.32 are small [30]. We calculated Cohen’s *d* for all participants, participants who completed the intervention, and participants with severe baseline symptoms.

##### Significant Improvement

We calculated significant improvement as described by Jacobson and Truax [31]. We subtracted the pre-test score from the post-test score and divided the difference by its standard error. All participants falling below 1.96 (or above for MBI-PA and EQ-5D) were considered significantly improved since this amount of change is unlikely to occur by chance (*P* < .05). The differences in improvement rate between the intervention and control group were then calculated with binary logistic regression and expressed as odds ratios (ORs) and their 95% confidence intervals (95% CIs).

##### Recovery

A different definition of recovery was used for the different types of outcome. The definitions were as follows: (1) depression—no DSM-IV diagnoses of major depression according to the MDI, (2) anxiety—a HADS score lower than 8 (a score ≥ 8 is indicative of a general anxiety disorder [32], and (3) work-related stress—not meeting the burnout criteria of the MBI. This was calculated only for those participants who did meet these criteria at baseline. The differences in recovery rate between the intervention and control group were also calculated with binary logistic regression and expressed as odds ratios and their 95% confidence intervals.

## Results

### Response Rates

Out of 213 enrolled participants, 177 filled in the post-test questionnaires (response rate 83.1%). The response was significantly higher in the control group (91%; n = 96) than in the intervention group (76%, n = 81; *P* = .004). Furthermore, the response was higher among the more educated participants (94.9%; n = 111) than among less educated participants (69%, n = 66; *P* < .001) and higher among participants without alcohol problems (87.1%, n = 121) than among those with alcohol problems (76%, n = 56; *P* = .04).

All the baseline differences between responders and nonresponders on the outcome measures were in the same direction: nonresponders reported poorer health at baseline than responders. However, the differences were very small and not statistically significant (Table 1).

**Table 1:** Baseline scores of depression, anxiety, burnout, and quality of life (N = 213)

Scale	Responders (n = 177), Mean (SD)	Dropouts (n = 36), Mean (SD)	*P* Value
CES-D	29.8 (9.3)	30.2 (8.6)	.80
MDI	24.3 (9.1)	26.7 (10.2)	.16
SCL-A	23.8 (7.1)	24.7 (8.1)	.47
HADS	10.0 (3.2)	10.1 (3.6)	.93
MBI-EE	2.8 (1.4)	2.9 (1.3)	.76
MBI-PA	3.2 (1.0)	3.6 (1.2)	.12
MBI-DP	2.4 (1.4)	2.2 (1.4)	.60
EQ-5D	0.62 (0.23)	0.61 (0.25)	.81

### Descriptive Analysis of Baseline Variables

As shown in Table 2, most participants in this study were female (71.4%; n = 152), born in the Netherlands (91.5%; n = 195), higher educated (54.9%; n = 117), and had a paid job (64.8%; n = 138). The participants in the intervention group were more often married (59.8%; n = 64) than participants in the control group (44.3%; n = 47; *P* = .02). There were no differences between the intervention and control groups with regard to baseline depression, anxiety, stress, or quality of life scores (Table 3).

**Table 2:** Baseline characteristics of the participants

Characteristic	All (N = 213), No. (%)	Intervention (N = 107), No. (%)	Control (N = 106), No. (%)	*P* Value
**Gender**				.85
Male	61 (28.6)	30(28.0)	31 (29.2)	
Female	152 (71.4)	77 (72.0)	75 (70.8)	
**Married**				.02
No	102 (47.9)	43 (40.2)	59 (55.7)	
Yes	111 (52.1)	64 (59.8)	47 (44.3)	
**Country of birth**				.31
Netherlands	195 (91.5)	100 (93.5)	95 (89.6)	
Other	18 (8.5)	7 (6.5)	11 (10.4)	
**Education**				.19
Lower	96 (45.1)	53 (49.5)	43 (40.6)	
Higher^*^	117 (54.9)	54 (50.5)	63 (59.4)	
**Paid job**				.85
No	75 (35.2)	37 (34.6)	38 (35.8)	
Yes	138 (64.8)	70 (65.4)	68 (64.2)	
**Sick leave^†^**				.32
No	111 (80.4)	54 (77.1)	57 (83.8)	
Yes	27 (19.6)	16 (22.9)	11 (16.2)	
**Alcohol problems**				.36
CAGE^‡^ < 2	139 (65.3)	73 (68.2)	66 (62.3)	
CAGE ≥ 2	74 (34.7)	34 (31.8)	40 (37.7)	
**Age, mean (SD)**	45.2 (10.6)	45.1 (10.9)	45.4 (10.4)	.84

^*^Higher education equals higher vocational education or university.

^†^Calculated only for the 64.8% (n = 138) participants with a paid job.

^‡^The CAGE questionnaire is a screening test for alcohol dependence.

### Adherence and Attrition

Of all 107 participants in the intervention group, 9% (n = 10) dropped out before the course started. The first assignment (Week 1) was completed by the remaining 91% (n = 97). Then another 17% (n = 18) dropped out, and the second assignment (Week 2) was completed by 74% (n = 79). Another 8% (n = 9) dropped out, and the third assignment (Week 3) was completed by 65% (n = 70). Finally, another 10% (n = 11) dropped out, leaving 55% (n = 59) who completed the whole course. Married participants more often completed the course (66%; n = 42) than non-married participants (40%, n = 17; *P* = .008). There were no other significant demographic or baseline differences between the participants who did or did not complete the course.

### Mean Improvements Scores: Depression, Anxiety, Stress, and Quality of Life

In general, the intervention had a significant effect on symptoms of depression, anxiety, and quality of life but not on work-related stress (Table 3). The analyses of all participants showed the most profound effects for the CES-D (*d* = 0.50) and the SCL-A (*d* = 0.42). In general, the effect sizes were largest for those participants who fully completed the intervention (n = 59). For these, the intervention was most effective for depression (CES-D: *d* = 0.67; MDI: *d* = 0.56), but the results for anxiety (SCL-A: *d* = 0.51; HADS: *d* = 0.48) and quality of life (EQ-5D: *d* = 0.44) were also substantial.

In a subset analysis, we selected only the participants with the most severe problems at baseline and calculated their improvements for each measure (Table 4). Compared to all participants (see Table 3), those with the most severe problems at baseline improved more, as evidenced by higher effect sizes, with the exception of scores on the SCL-A scale, for which the effect size decreased from 0.42 to 0.37. Improvements in effect size were most notable for work-related stress: the overall effect size on the MBI-EE subscale was 0.28 for all participants but improved to 0.65 for participants who actually experienced a burnout at baseline.

**Table 3:** Effects of self-examination therapy on depression, anxiety, burnout, and quality of life

Scale^*^	Control (N = 106), Mean (SD)		Intervention, All (N = 107), Mean (SD)		Intervention, Course Completers (N = 59), Mean (SD)		Effect Size^†^ (95% CI)	
	Pre-Test	Post-Test	Pre-Test	Post-Test	Pre-Test	Post-Test	All	Course Completers
CES-D	29.9 (9.2)	26.2 (10.5)	29.9 (9.1)	20.9 (10.8)	29.8 (8.5)	19.3 (10.1)	0.50 ( 0.22-0.79)	0.67 (0.32-1.02)
MDI	23.6 (9.0)	25.1 (6.8)	25.8 (9.6)	22.9 (6.9)	25.1 (8.9)	21.4 (6.2)	0.33 ( 0.03-0.63)	0.56 (0.22-0.90)
SCL-A	23.7 (7.2)	22.7 (7.5)	24.1 (7.4)	19.7 (6.8)	10.0 (2.9)	19.1 (6.2	0.42 ( 0.14-0.70)	0.51 (0.18-0.84)
HADS	9.9 (3.3)	9.1 (3.3)	10.1 (3.3)	8.0 (3.4)	24.2 (7.0)	7.5 (3.2)	0.33 ( 0.04-0.61)	0.48 (0.15-0.82)
MBI-EE	2.8 (1.5)	2.8 (1.5)	2.9 (1.3)	2.5 (1.5)	2.8 (1.1)	2.5 (1.4)	0.28 (−0.08 to 0.64)	0.20 (−0.26 to 0.66)
MBI-PA	3.4 (1.0)	3.2 (1.0)	3.2 (1.1)	3.5 (1.0)	2.2 (1.3)	3.5 (1.0)	0.33 (−0.03 to 0.69)	0.36 (−0.25 to 0.98)
MBI-DP	2.4 (1.4)	2.6 (1.5)	2.4 (1.3)	2.3 (1.4)	3.1 (1.2)	2.2 (1.5)	0.20 (−0.15 to 0.56)	0.27 (−0.22 to 0.75)
EQ-5D	0.61 (0.24)	0.66 (0.20)	0.62 (0.23)	0.73 (0.20)	0.63 (0.22)	0.8 (0.2)	0.31 ( 0.03-0.60)	0.44 (0.11-0.77)

^*^The values for the MBI subscales are only given for those with a paid job; n = 70 in the intervention condition; n = 68 in the control.

^†^Effect size is presented as Cohen’s *d*: the number of standard deviations the intervention group has improved more than the control group; (M_c_ – M_i_) / Sd_ic_.

**Table 4:** Effects of self-examination therapy on the subset of participants with severe symptoms of depression, anxiety, burnout, and quality of life at baseline

Scale	Definition of Severe Symptoms	Control			Intervention			Effect Size^*^ (95% CI)
		No.	Pre-Test, Mean (SD)	Post-Test, Mean (SD)	No.	Pre-Test, Mean (SD)	Post-Test, Mean (SD)	
CES-D	≥ 16	99	31.1 (8.1)	27.3 (9.8)	97	31.6 (7.6)	21.7 (10.8)	0.54 (0.25-0.84)
MDI	DSM-IV depression	37	32.8 (5.2)	28.3 (6.9)	44	33.7 (5.5)	25.5 (6.8)	0.41 (−0.04 to 0.86)
SCL-A	≥ 18	89	25.5 (6.4)	24.1 (7.3)	84	26.7 (6.0)	21.6 (6.4)	0.37 (0.06-0.69)
HADS	≥ 8	78	11.3 (2.5)	10.2 (3.0)	85	11.3 (2.6)	8.7 (3.3)	0.45 (0.13-0.78)
MBI-EE	burnout	34	3.9 (1.0)	3.8 (1.3)	43	3.4 (1.0)	2.9 (1.3)	0.65 (0.14-1.16)
MBI-PA	burnout	34	3.1 (0.9)	3.0 (0.9)	43	2.9 (1.0)	3.3 (1.1)	0.33 (−0.14 to 0.81)
MBI-DP	burnout	34	3.3 (1.1)	3.2 (1.4)	43	2.9 (1.3)	2.6 (1.5)	0.44 (−0.06 to 0.95)
EQ-5D	≥ 0.55	74	0.75 (0.06)	0.7 (0.2)	73	0.76 (0.08)	0.8 (0.2)	0.34 (0.00-0.69)

^*^Effect size is presented as Cohen’s *d*: the number of standard deviations the intervention group has improved more than the control group; (M_c_ – M_i_) / Sd_ic_.

### Significant Improvement

The proportion of participants with significant improvements (their change is so large it is unlikely to have occurred by chance, see definition under “Methods”) in both groups is compared in Table 5. The results show significant effects of the intervention both for depression (CES-D and MDI) and anxiety (SCL-A and HADS). The improvements on the MBI-PA scale are also statistically significant. The differences between the intervention and the control groups for the remaining outcomes were all in favour of the intervention group (OR between 1.6 and 2.2), but these results were not statistically significant.

**Table 5:** Participants with significant improvement

Scale	Intervention (N = 107), No. (%)	Control (N = 106), No. (%)	OR	95% CI
CES-D	52 (48.4)	22 (20.9)	3.5	1.9-6.7
MDI	22 (20.7)	7 (6.6)	3.7	1.4-10.0
SCL-A	23 (21.3)	12 (11.3)	2.1	1.0-4.6
HADS	38 (35.9)	16 (15.5)	3.1	1.6-6.0
MBI-EE	14 (13.4)	7 (6.8)	2.2	0.6-8.1
MBI-PA	23 (21.4)	7 (6.5)	3.9	1.2-12.6
MBI-DP	8 (7.7)	5 (4.7)	1.7	0.4-7.1
EQ-5D	25 (23.7)	17 (16.2)	1.6	0.8-3.3

### Recovery

Of all 81 participants who suffered major depression according to the MDI at baseline, a total of 52 (64.4%) had recovered at post-test across both groups (Table 6). Recovery occurred more often in the intervention group (72.7%) than in the control group (54.6%, OR = 2.2), but this effect was not statistically significant (95% CI 0.8-6.0). Recovery from anxiety and burnout also occurred more often in the intervention group than in the control group. However, the result with regard to anxiety was not statistically significant (OR = 2.0; 95% CI 0.9-4.2), while that for burnout was (OR = 4.0; 95% CI 1.2-13.5).

**Table 6:** Recovery of participants with depression, anxiety, and burnout (as established at baseline)

	Total No. Participants at Baseline	Definition of Recovery	Post-Test, No. (%)		OR	95% CI
			Intervention	Control		
Depression	81	No MDI diagnoses	32/44 (72.7)	20/37 (54.6)	2.2	0.8-6.0
Anxiety	134	HADS < 8	26/70 (37.7)	15/64 (23.4)	2.0	0.9-4.2
Burnout	77	No MBI diagnosis	16/43 (38.1)	5/34 (13.5)	4.0	1.2-13.5

## Discussion

### Principal Results

We studied the effects of a short, generic, Web-based, self-help intervention for mental health problems in a randomized trial among 213 participants with symptoms of depression, anxiety, or work-related stress. We demonstrated statistically and clinically significant effects on symptoms of depression and anxiety. These effects were even more pronounced among participants with more severe baseline problems and for participants who fully completed the course. The effects on work-related stress and quality of life were less clear.

### Limitations

This study has several limitations. The first is related to the choice of the control group. We could have chosen a care-as-usual comparison (ie, not have given any intervention to the control group); however, this might have limited the generalizability of our results since in that case only patients willing to be randomized to a non-treatment option would have participated. It is likely that these patients differ from the ones who do want (need) treatment. We also might have chosen an attention placebo control group or comparison with another intervention. It is known that effects of attention placebo controlled trials are usually smaller than waitlist controlled trials. However, with our intervention, we especially intended to reach those people who do not get any treatment at all [33], and in this case, we feel that a comparison with a waiting list control group is justified. We stress that the demonstrated effects might, in part, be caused by common therapy factors and not by the specific intervention we studied (eg, the attention given to the intervention group by means of email support might have caused effects regardless of the contents of the feedback or the intervention). Nevertheless, since we intend to implement the course in the Netherlands as is (including support), this effect is what we wanted to measure.

The second limitation has to do with the response rate. Although the overall response rate was satisfactory (83%), the response rate of the intervention group was significantly lower (76%) than that of the control group (91%). We could find no indications for selection bias since we could not demonstrate clear baseline differences between the responders and nonresponders (except for marital status). The bias that still might have been introduced was accounted for by imputing all missing data (multiple imputations) and performing intention-to-treat analyses. Nevertheless, imputing 24% of the data might have led to unreliable estimates.

Another limitation is the fact that participants could only be included in the study if they had computer skills and access to Internet. Thus, the participants in this study were more highly educated than the general population, and it is uncertain whether the results of this study can be generalized to people with less education.

### Specific Findings

Meta-analyses for bibliotherapy regarding different types of target problems have shown effect sizes between 0.53 and 0.96 [3]. For depression and anxiety, a recent meta-analysis of Web-based, cognitive behavioral, self-help interventions showed mean effect sizes (Cohen’s *d*) of 0.32 and 0.96, respectively [5]. Thus, our results on symptoms of depression and anxiety seem to fit well within the reported range. It is important to note that our results were obtained in less time (4 weeks) than is usual for Web-based interventions for anxiety or depression (often 6 weeks or more). Furthermore, our results are also almost identical to those found in a meta-analysis of face-to-face problem-solving treatment (*d* = 0.42) [34]. All this implies that our intervention may be a worthwhile alternative to other more intensive or expensive treatment options, especially since it can be used for participants with comorbid symptoms of anxiety and depression. However, longer follow-up studies are necessary to determine the treatment gains over a longer period of time.

The results with regard to work-related stress were less consistent. When considering only those participants who were suffering from burnout at the start of the study, the results were promising. The participants in the intervention group were four times (95% CI 1.2-13.5) more likely to recover from their burnout than participants in the control group, and they experienced a substantial improvement with regard to the EE subscale of the MBI (Cohen’s *d* = 0.65). These effects disappeared when considering all participants (or all participants who completed the intervention). This probably can be explained by the relatively small percentage of participants who actually did experience work-related stress at the start of the study: only 77 participants (36%) could be described as suffering from burnout. Furthermore, it must be noted that, in general, the effects of interventions for work-related stress seem to be less pronounced. Meta-analyses have reported effect sizes between 0.35 and 0.68 for different types of face-to-face intervention [35].

### Conclusion

To our knowledge, this is the first trial on a short, Web-based, problem-solving intervention for participants with different types of (comorbid) emotional problems. The results seem to be as good as other longer, disease-specific bibliotherapies. Longitudinal research is needed to study the long-term effects.

## Multimedia Appendix 1

Intervention screenshots

## Abbreviations

CES-D: Center for Epidemiological Studies Depression Scale
DSM-IV: Diagnostic and Statistical Manual of Mental Disorders, 4th Edition
EQ-5D: EuroQoL questionnaire
HADS: Hospital Anxiety and Depression Scale (only Anxiety section is used)
MDI: Major Depression Inventory
MBI: Maslach Burnout Inventory
MBI-DP: MBI Depersonalization subscale
MBI-EE: MBI Emotional Exhaustion subscale
MBI-PA: MBI Personal Accomplishment subscale
SCL-A: Symptom Checklist – Anxiety section
