# Supplementary material for: Effectiveness of a Web-Based Self-Help Intervention for Symptoms of Depression, Anxiety, and Stress: Randomized Controlled Trial
Source: J Med Internet Res. 2008 Mar 25;10(1):e7. doi: 10.2196/jmir.954 (PMC2483843; doi:10.2196/jmir.954)
Supplement: Supplementary file 1 [file jmir_v10i1e7_app1.pdf]

Leeromgeving

http://allesondercontrole.psy.vu.nl:8080/didactor/education/index.jsp?provider=331&education=827&cla

Google

login | instellingen | print | ueelgestelde uragen | e-mail begeleider

Alles onder controle

Lessen

start lessen resultaten

vooraf | **les 1** | les 2 | les 3 | les 4 | les 5 | les 6

les 1

wat is echt belangrijk | zorgen en problemen | wel of niet oplosbaar

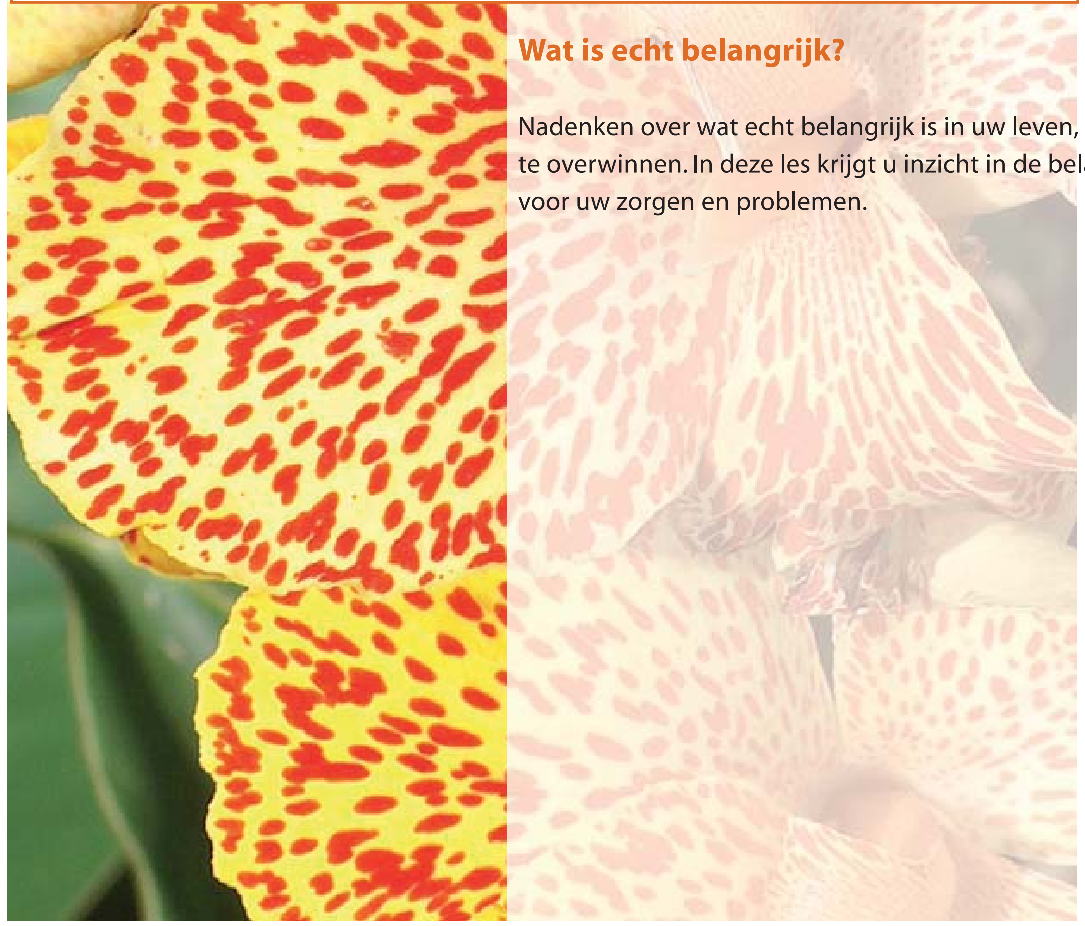

Wat is echt belangrijk?

Nadenken over wat echt belangrijk is in uw leven, kan helpen om uw zorgen, spanningen, somberheid en angsten te overwinnen. In deze les krijgt u inzicht in de belangrijke dingen in uw leven. Ook ontdekt u wat dat betekent voor uw zorgen en problemen.

Leeromgeving

http://allesondercontrole.psy.vu.nl:8080/didactor/education/index.jsp?provider=331&education=827&cla

login | instellingen | print | veelgestelde vragen | e-mail begeleider

Alles onder controle

Lessen

start lessen resultaten

vooraf | **les 1** | les 2 | les 3 | les 4 | les 5 | les 6

les 1

wat is echt belangrijk | zorgen en problemen | wel of niet oplosbaar

Wat vind ik belangrijk?

Nadenken over wat u echt belangrijk vindt, kan helpen om uw zorgen, spanningen, somberheid en angsten te overwinnen. De komende vijf weken neemt u elke week een half uur de tijd om op een rij te zetten wat voor u de belangrijkste dingen in uw leven zijn. Of dat nu in uw persoonlijke leven is, uw werk, maatschappelijk leven of vrijetijdsbesteding. Denk bijvoorbeeld aan: een goede gezondheid, uw kinderen, uw carrière. Of: een bijdrage leveren aan een gezonder milieu. Of een waardevolle bijdrage leveren aan de samenleving. Bekijk ook eens de voorbeelden van uw medecursisten.
De eerste keer dat u dit doet, is het moeilijkst. Daarna is het een kwestie van aanscherpen. Bedenk elke keer: is dit echt het belangrijkste voor mij? Zodat u na vijf weken zeker weet: dit is waar het voor mij om gaat in het leven.

Tips:
- Neem de tijd. De eerste keer heeft u misschien wat langer nodig. Stop na een uur.
- Het gaat om wat ú belangrijk vindt. Laat u niet beïnvloeden door wat anderen vinden. Of door wat u denkt dat hoort.
- Maak de lijst niet te lang. Schrijf alleen de allerbelangrijkste dingen op. De meeste mensen komen op zo'n vijf punten.

Maak een lijst van dingen die u belangrijk vindt in uw leven.

toelichting

opslaan | annuleer

Leeromgeving

http://allesondercontrole.psy.vu.nl:8080/didactor/education/index.jsp?provider=331&education=827&cla

login | instellingen | print | veelgestelde vragen | e-mail begeleider

Alles onder controle

Lessen

start lessen resultaten

vooraf | **les 1** | les 2 | les 3 | les 4 | les 5 | les 6

les 1

wat is echt belangrijk | zorgen en problemen | wel of niet oplosbaar

Wat vind ik belangrijk?

Nadenken over wat u echt belangrijk vindt, kan helpen om uw zorgen, spanningen, somberheid en angsten te overwinnen. De komende vijf weken neemt u elke week een half uur de tijd om op een rij te zetten wat voor u de belangrijkste dingen in uw leven zijn. Of dat nu in uw persoonlijke leven is, uw werk, maatschappelijk leven of vrijetijdsbesteding. Denk bijvoorbeeld aan: een goede gezondheid, uw kinderen, uw carrière. Of: een bijdrage leveren aan een gezonder milieu. Of een waardevolle bijdrage leveren aan de samenleving. Bekijk ook eens de voorbeelden van uw medecursisten.
De eerste keer dat u dit doet, is het moeilijkst. Daarna is het een kwestie van aanscherpen. Bedenk elke keer: is dit echt het belangrijkste voor mij? Zodat u na vijf weken zeker weet: dit is waar het voor mij om gaat in het leven.

Tips

- Neem de tijd. De eerste keer heeft u misschien wat langer nodig. Stop na een uur.
- Het gaat om wat ú belangrijk vindt. Laat u niet beïnvloeden door wat anderen vinden. Of door wat u denkt dat hoort.
- Maak de lijst niet te lang. Schrijf alleen de allerbelangrijkste dingen op. De meeste mensen komen op zo'n vijf punten.

Maak een lijst van dingen die u belangrijk vindt in uw leven.

toelichting

opslaan | annuleer

Leeromgeving

http://allesondercontrole.psy.vu.nl:8080/didactor/education/index.jsp?provider=331&education=827&cla

Google

login | instellingen | print | veelgestelde vragen | e-mail begeleider

Alles onder controle

lessen

start lessen resultaten

les 1

vooraf | **les 1** | les 2 | les 3 | les 4 | les 5 | les 6

wat is echt belangrijk | **zorgen en problemen** | wel of niet oplosbaar

Zorgen en problemen

Wie depressief is, wordt vaak zo in beslag genomen door zijn zorgen en problemen dat hij ze niet meer de baas kan. In deze cursus leert u hoe u uw zorgen weer onder controle krijgt.
Deze week maakt u een lijst van alles wat u dwarszit, waarover u zich zorgen maakt, of waarover u zich gespannen, depressief of angstig voelt. Schrijf alles op wat u kunt bedenken. Het maakt niet uit of het probleem klein of groot is. Wees zo volledig mogelijk. Doe dit een week lang elke dag. Neem per dag tien minuten de tijd.

Maak een lijst van alles wat u dwarszit, wat uw zorgen zijn en waarover u zich angstig, depressief of gespannen voelt.

opslaan | annuleer

Leeromgeving

http://allesondercontrole.psy.vu.nl:8080/didactor/education/index.jsp?provider=331&education=827&cla

Alles onder controle

lessen

les 1

Wel of niet oplosbaar?

Problemen zijn globaal in te delen in drie soorten. We nemen de belangrijkste kort even door.

Niet belangrijk of niet relevant: De problemen of zorgen die niets te maken hebben met wat belangrijk voor u is, zijn meestal ook niet belangrijk genoeg om u mee bezig te houden. Toch is het soms moeilijk om deze problemen of zorgen van u af te zetten. Hoe u daarmee kunt omgaan, komt bij les 2 aan de orde.

Belangrijk en oplosbaar: Belangrijke problemen en zorgen hebben meestal te maken met dingen die belangrijk zijn in uw leven. Veel van die problemen zijn oplosbaar, ook al is de oplossing niet altijd even makkelijk te bereiken. Hoe u met oplosbare problemen aan de slag kunt, leest u in les 2.

Belangrijk en onoplosbaar: Er zijn ook belangrijke problemen die niet op te lossen zijn. Bijvoorbeeld de dood van een familielid. Of een ziekte. In les 2 leest u wat helpt om dergelijke gebeurtenissen een plek te geven in uw leven.

Na een week is uw lijst met zorgen en problemen compleet. Nu is het zaak de problemen onder te verdelen in: onbelangrijk, belangrijk en oplosbaar, en belangrijk en onoplosbaar.

Bekijk uw lijst met zorgen en porblemen. Geef aan welk soort probleem het si en hoeveel last u ervan heeft.

Ik kan het allemaal niet aan	soort probleem	hoeveel last
Ik ben zo vergeetachtig	soort probleem	hoeveel last
Ik mis mij pas overleden moeder	soort probleem	hoeveel last
De wasmachine is kapot	soort probleem	hoeveel last

opslaan | annuleer
